# Shame and Guilt in Social Anxiety Disorder: Effects of Cognitive Behavior Therapy and Association with Social Anxiety and Depressive Symptoms

**DOI:** 10.1371/journal.pone.0061713

**Published:** 2013-04-19

**Authors:** Erik Hedman, Peter Ström, Angela Stünkel, Ewa Mörtberg

**Affiliations:** 1 Department of Clinical Neuroscience, Osher Center for Integrative Medicine, Karolinska Institutet, Stockholm, Sweden; 2 Department of Clinical Neuroscience, Division of Psychology, Karolinska Institutet, Stockholm, Sweden; 3 Department of Psychology, Stockholm University, Stockholm, Sweden; West China Hospital of Sichuan University, China

## Abstract

Social anxiety disorder (SAD), characterized by fear of being scrutinized by others, has features that that are closely linked to the concept of shame. Despite this, it remains to be investigated whether shame is elevated in persons with SAD, and if cognitive behavior therapy (CBT) for SAD could reduce shame experience. In the present study, we focused on internal shame, i.e. the type of shame that pertains to how we judge ourselves. Although guilt is distinctly different from shame, we also viewed it as important to investigate its role in SAD as the two emotions are highly correlated. The aim of this study was to investigate: (I) if persons with SAD differ from healthy controls on shame and guilt, (II) if shame, guilt, depressive symptoms, and social anxiety are associated in persons with SAD, and (III) if CBT can reduce internal shame in patients with SAD. Firstly, we conducted a case-control study comparing a sample with SAD (n = 67) with two samples of healthy controls, a main sample (n = 72) and a replication sample (n = 22). Secondly, all participants with SAD were treated with CBT and shame, measured with the Test of Self-Conscious affect, was assessed before and after treatment. The results showed that shame was elevated in person with SAD compared to the control replication sample, but not to the main control sample. In addition, shame, social anxiety, and depressive symptoms were significantly associated among participants with SAD. After CBT, participants with SAD had significantly reduced their shame (Cohen's *d* = 0.44). Guilt was unrelated to social anxiety. We conclude that shame and social anxiety are associated and that it is likely that persons with SAD are more prone to experience shame than persons without SAD. Also, CBT is associated with shame reduction in the treatment of SAD.

## Introduction

Social anxiety disorder (SAD) is characterized by a debilitating fear of being scrutinized by others and avoidance of social events that evoke this fear [1]. It is the one of the most common psychiatric disorders and associated with substantial impairment and an increased risk of developing other psychological problems [2], [3]. Many features of SAD, such as fear of speaking in front of an audience, are highly prevalent also in the normal population [4]. From an evolutionary perspective social anxiety probably plays an important role in preventing the individual to act in ways that could put them at risk of being excluded from the social community, and has therefore been described as the fear of exclusion [5].

Several aspects of social anxiety and SAD are linked to the concept of shame. Shame can be defined as an affect involving perceptions that others view oneself as having negative personal characteristics or that one has engaged in behaviors that are unattractive [6], [7]. This affective state is made possible by the human complex ability of imagining how one is represented in the minds of others and could be viewed as a signal that one is at risk of losing social rank or being rejected [8]. The ultimate function of shame is to motivate behaviors that are appealing to others, signal submission, and limit possible social damage [9], [10]. The similarities of shame and guilt are often underscored, but there is a clear distinction between the two [6], [11]. Guilt is largely unrelated to the perception of others and relates to specific actions that violates others' rights according to one's own view, and promotes remorse and restorative behaviors [12]. Shame on the other hand, is entirely related to one's perceived attractiveness, and is to a larger extent therefore directed towards the self rather than to specific behaviors [12]. An important aspect of shame is that it can be external or internal [13]. External shame refers to the affect that is based on how one is perceived by others. This is sometimes referred to as stigma awareness and concerns aspects of one's actions that could lead to rejection or criticism, if they were known to others [13]. Internal shame could be defined as shame based on how the individual views him- or herself. As expressed by Matos et al. [10], internal shame refers to when we are both the judge and the judged. As pointed out by Gilbert [14] internal and external shame are not always correlated as it is possible to engage in behaviors that are externally shameful, e.g. xenophobic behaviors, but that do not lead to internal shame. That is, they do not lead to self-criticism or feelings of inadequacy when performed. Of course, the reversed pattern is also possible, i.e. when external shame is absent and one is perceived as attractive by others but one at the same time has a strong negative self-perception that leads to internal shame [14]. However, empirical data suggest that this latter pattern is more uncommon meaning that if internal shame is high, then external shame is also likely to be high [15].

When comparing a broad definition of shame to the preeminent cognitive models of social anxiety [16], [17] there seems to be major overlaps. According to Clark and Wells [16], a core feature of social anxiety is the strong desire to appear favorable to others but believing that one is incapable of doing so. Other social anxiety theorists like Rapee and Heimberg [17], describe similar central processes to be at the heart of social anxiety. They stress the importance of how the individual believes he or she is perceived by the audience and that social anxiety occurs when one makes the judgment that one fails to meet the demands of others [17]. Thus, models of social anxiety clearly involve processes similar to shame if defined, as the affect arising from the belief that one is unattractive in the eyes of others. However, this overlap of constructs holds primarily for an external definition of shame. As for internal shame, the association with social anxiety is much less obvious both from a theoretical and an empirical perspective. The cognitive models of social anxiety clearly emphasize the importance of how the individual thinks he or she is perceived by others and do not predict stable negative self-evaluations in persons with social anxiety disorder [16], [17]. In fact, Clark and Wells [16] specifically mention that assumptions about the self are different in socially anxious individuals compared to depressed persons as the former group generally does not view themselves as inadequate in general, but that negative perceptions are mainly related to the feared social situation. Persons with depression, on the other hand, are more likely to have stable assumptions or schemata regarding negative self-evaluation [16].

Prior literature investigating the association of social anxiety to internal shame suggests a moderate association. Studies by Fergus et al. [11] and by Gilbert [7] showed that internal shame as measured by the Test of Self-Conscious Affect (TOSCA) and social anxiety were significantly correlated with *r*s of .52–.54. The same magnitude of correlations were found in a recent study by Matos et al. [10], which also showed that internal and external shame were moderately associated. In these studies, guilt was unrelated to psychiatric symptom burden leading to the conclusion that shame-free guilt probably has several highly adaptive functions [7], [11]. In the study by Fergus et al. [11] there was even a trend towards negative association of guilt and social anxiety. The absence of association between social anxiety and guilt is expected from a theoretical perspective, as it plays no role in the dominant models of social anxiety. To our knowledge, very little data has been published on the relationship between internal shame and SAD. Although one study used data partly collected from persons with SAD [11], sample estimates were based on a pooled group comprising participants with different anxiety disorders making it difficult to assess the role of shame in SAD specifically.

SAD can be effectively treated with cognitive behavior therapy (CBT), which has been shown in more than 20 randomized controlled trials [18]. However, research on whether the CBT has an effect on internal shame in the treatment of SAD is scarce. One study showed that persons with an anxiety disorder undergoing intensive exposure-based treatment for two to three weeks made improvements in social anxiety and that these improvements were correlated with reductions of shame [11]. However, in that study there was no separate report on SAD and we have found no studies investigating the effect of individual CBT for SAD on shame. In addition, no prior study has investigated whether the treatment format, i.e. individual or group format, moderates the association of shame and outcome in CBT for SAD. More knowledge in this regard could ultimately lead to a better understanding of social anxiety disorder and how CBT achieves its effect in the treatment of SAD.

In summary, shame and social anxiety have common features, but this association is stronger for external than for internal shame. The former is characterized by perceptions of unattractiveness in the eyes of others while the shame experience in the latter sense is marked by negative self-evaluations. Guilt is often discussed as an affect similar to shame, but evidence does not suggest that experience of guilt is related to psychopathology. The role of shame in patients with SAD is poorly investigated and it is unclear whether internal shame is elevated in these persons. In addition, the knowledge on the effects of CBT on internal shame is scarce.

The aim of this study was to investigate the association of internal shame, guilt, depressive symptoms, and social anxiety. We collected data from persons with SAD before and after CBT and from healthy controls. The specific questions that we sought to answer were:

- Do persons with SAD differ from healthy controls on measures of internal shame and guilt?
- Are internal shame, guilt, depressive symptoms, and social anxiety associated in persons with SAD?
- Can CBT reduce internal shame in patients with SAD?

We hypothesized that internal shame, but not guilt, would be moderately associated with social anxiety. We also expected that CBT would reduce shame in patients with SAD.

## Methods

### Design

To investigate the association of shame and social anxiety two types of comparisons were made. First, we compared the experience of shame using a case-control design in a sample of patients with SAD (n = 67) to a sample of healthy controls (main control sample, n = 72). After analysis of these data, a second sample of healthy control participants was recruited (n = 22) to serve as a cross validation sample. The latter sample was better matched on demographic characteristics and is denoted the replication sample. Second, we investigated the extent to which measures of internal shame, guilt, depressive symptoms and social anxiety were correlated among the patients with SAD.

To investigate the effect of CBT on internal shame, a within-group pretest-posttest design was used where the patients with SAD were assessed on measures of shame directly before treatment and at one-year follow-up. Participants received CBT based on the treatment developed by David M. Clark and colleagues [19]. The treatment was delivered either in an individual format (n = 32) or as a group therapy (n = 35). Participants received treatment within the context of a randomized controlled trial (RCT). The main results of that study have been reported elsewhere [20].

### Recruitment procedure and participants

#### SAD participants

The SAD patients were recruited from an outpatient clinic as part of an RCT. The main inclusion criteria were that participants had to have a principal diagnosis of SAD according to DSM-IV as assessed using the SCID [21], be between 18 and 65 years of age, and have no history of bipolar or psychotic disorder. Table 1 provides data on demographic characteristics of the sample.

**Table 1:** Demographic description of the participants.

Variable		SADSample	Healthy controls, main sample(HC-M)	Healthy controls, replication sample(HC-R)	Test statisticSAD vs. HC-M	Test statisticSAD vs. HC-R
		n = 67	n = 72	n = 22		
**Gender**	Women (%)	43 (64.2)	58 (80.6)	13 (58.1)	*χ^2^* = 4.89;df = 1*p*<.03*	*χ^2^* = 0.18df = 1*p*<.80
	Men (%)	24 (35.8)	14 (19.4)	9 (40.9)		
**Age**	Mean age (SD)	33.5 (9.1)	25.8 (6.3)	32.6 (10.7)	*t* = 5.74;df = 1,137*p*<.001*	*t* = 0.41df = 1, 87*p*<.71
	Min-max	19–55	19–49	22–54		
**Social anxiety disorder**	Generalized subtype (%)	46.0 (68.6)	Notapplicable	Notapplicable		
	Mean duration, years	19.4				
	Mean age of onset	14.8				
**Occupational status**	Student (%)	11 (16.4)	72 (100.0)	22 (100)		
	Employed (%)	44 (65.7)	0 (0.0)	0 (0.0)		
	Unemployed (%)	5 (7.4)	0 (0.0)	0 (0.0)		
	Sick leave (%)	7 (10.4)	0 (0.0)	0 (0.0)		

*Abbreviations:* SAD, social anxiety disorder; HC-M, Healthy controls-main sample; HC-R, Healthy controls, replication sample;

* = significant at alpha-level .05.

#### Healthy controls

The healthy controls were students at the department of psychology at Stockholm University, Sweden. Of 118 assessed potential healthy controls 24 were excluded due to elevated social anxiety scores on a screening test with the Mini Social Phobia Inventory [Mini-Spin; 22]. Healthy controls were recruited at two separate occasions. There were 72 participants in the main control sample and 22 participants in the replication sample, i.e. in the sample recruited after the first analyses had been conducted on the main control sample. As presented under “Statistical analyses”, the power to detect a difference between the clinical sample and the healthy control groups was considered adequate. Thus, there were 94 controls in total. The replication sample was recruited among students at the psychology/psychotherapist program in order to better match the demographic characteristics of the SAD participants. Control participants provided assessments at one occasion only and did not receive treatment with CBT. Demographic data on the sample is presented in Table 1. As shown in Table 1, there were significant differences between SAD participants and the healthy control main sample in terms of age and gender, i.e. the latter group was younger and comprised more women. However, there were no significant differences between the SAD participants and the healthy control replication sample on these variables.

### Measures

#### Shame and guilt

The Test of Self-conscious Affect (TOSCA) [23] was used to assess shame and guilt. The TOSCA also assesses externalization, pride and, detachment, but these constructs are not reported in the present paper. The instrument is comprised of a description of 15 different situations and the respondent rates to which extent he or she agrees with suggested potential reactions relating to shame and guilt. Each item response is scored on a Likert scale (1–5) and the total scale range for shame and guilt is 15 to 75. The shame scale of the TOSCA primarily measures internal shame and not external [7]. As described by Gilbert [7], the scale consists of items relating to self-evaluation (feeling stupid), shame behaviors (avoid eye contact), and affect (self-disgust). The subscales measuring shame and guilt have been shown to possess good psychometric properties including good test-retest reliability [shame scale, r = .84; guilt scale, r = .75; 24] and good internal consistency [shame scale, Cronbach's α = .84; guilt scale Cronbach's α = .74; 11].

#### Social anxiety

The Social Interaction anxiety Scale (SIAS) [25] and the Liebowitz Social Anxiety Scale-Self-report -SR (LSAS-SR) [26] were used to assess social anxiety. The scale range of the SIAS is 0 to 80 and the total score of the LSAS-SR is between 0 and 144. Both instruments have good psychometric properties including high test-retest reliability over 12 weeks (SIAS, *r* = .92; LSAS-SR, *r* = .82) [25], [26] and good internal consistency (SIAS, Cronbach's α = .83; LSAS-SR, Cronbach's α = .95) [25], [26]. The LSAS-SR and the SIAS were used solely as continuous measures of social anxiety and were administered independently of the diagnostic interview.

#### Depressive symptoms

We used the Beck Depression Inventory (BDI) [27] to assess depressive symptoms. The scale range of the BDI is 0 to 63 and the scale has been shown to have high test-retest reliability over two weeks (*r* = .78) [28] and high internal consistency (Cronbach's α = .93) [27].

#### Diagnostic assessment

To establish whether participants in the clinical sample met diagnostic criteria for SAD the Structured Clinical Interview for DSM-IV Axis I-disorders (SCID) [21] was used, which has been shown to have high inter-rater reliability (r = .86) [29]. To screen for SAD among the students that were under consideration to be included as healthy controls we used the Mini-Social Phobia Inventory (Mini-SPIN) [22]. The Mini-SPIN has been shown to be sensitive to detect SAD (90% precision) when using a cutoff of 6 on a scale from 0 to 12 [22], [30]. In the present study potential healthy controls who scored ≥6 were excluded from the study.

#### Cognitive behavior therapy

All participants with SAD received CBT based on the treatment protocol developed by David M. Clark and colleagues [19]. This treatment is based on the cognitive model as elaborated by Clark and Wells [16] and emphasizes the role of self- focused attention, safety behaviors and dysfunctional assumptions as maintaining factors of social anxiety. Participants in this study received CBT within the context of an RCT [31] and although all participants underwent CBT, half of the sample (n = 32) was randomized to receive it in individual format, while the other half (n = 35) was randomized to CBT in a group format. All CBT entailed the same components which were the following: (a) deriving an individualized version of the cognitive model using patients' thoughts, images, anxiety symptoms, safety-behaviors and attentional strategies, (b) conducting a behavioral experiment to demonstrate the adverse effects of safety behaviours, (c) using video feedback to modify distorted self-imagery, (d) training externally focused attention (i.e., to shift attention away from oneself and onto to the social situation), (e) conducting behavioral experiments to enable patients to test the validity of their negative predictions in a variety of social situations, (f) identification and modification of problematic anticipatory and post-event negative processing, and (g) identification and modification of dysfunctional assumptions. In the individual format participants received 16 weekly sessions. Group CBT was led by two therapists and comprised 17 sessions and there were 6–7 participants in each group. Group CBT was in an intensive format, which meant that all 17 group sessions were delivered in three weeks time. Seven therapists (five psychologists, one nurse and one psychiatrist) delivered the treatments. To facilitate treatment integrity, all therapists received supervision throughout the treatment period by an experienced clinical psychologist and sessions were videotaped and checked for integrity during supervision.

### *Assessment points and procedure*

Participants with SAD completed assessments with the TOSCA, LSAS-SR, SIAS, and the BDI before treatment (baseline) and at one-year follow-up. Healthy controls completed the TOSCA and the Mini-SPIN at one occasion. The RCT study was approved by the regional ethics review board in Stockholm and conducted in accordance with the guidelines of the Declaration of Helsinki. Participants in the clinical sample, i.e. participants with SAD, provided written informed consent and participants in the control condition verbal informed consent. Verbal informed consent for healthy controls was viewed as sufficient as these participants were exposed to minimal risk of adverse events. That is, they only completed two relatively brief self-report questionnaires anonymously, and underwent no psychiatric assessment or any form of intervention.

### Statistical analyses

All analyses were conducted using SPSS version 20.0 (IBM inc. Chicago). Continuous data were analyzed using Pearson correlations (zero-order and partial), linear regression models, and independent and paired samples t-tests. Nominal data were analyzed using *χ^2^* -tests. Analysis of patterns of missing data were conducted using Little's Missing Completely at Random Test [32]. Missing data were imputed using linear regression estimation methods using the available covariates as predictors. As the missing value analysis showed that data were missing completely at random and imputation of missing data had no significant effect on the obtained estimates, the presented results are based on observed data, i.e. without imputation of missing values. Power calculations showed that there was 80% probability of detecting a difference of moderate effect between the clinical sample and the main control sample (0.5 *d*) using an alpha-level of .05. The replication sample size was considered adequate as power to detect a large effect (*d≥*0.80) was 80% with an alpha-level of .05. Power to detect baseline to post-treatment differences (0.5 *d*) among participants with SAD was 99% with the same alpha-level. Effect sizes were, Cohen's *d*, were calculated based on pooled SDs.

## Results

### Attrition

In the control group there was no data loss. In the clinical sample, 62 out of 67 participants (93%) completed all assessments at baseline, while 48 out of 67 (72%) participants provided data at follow-up. Using Little's test missing values were found to be missing completely at random (*χ^2^*_(1655)_ = 757.4, *p*<.99).

### Shame and guilt - between group analyses at baseline

#### SAD vs. Healthy controls main sample

Means and SDs of the TOSCA shame and guilt scales are presented in Table 2. There was no significance difference between the participants with SAD at baseline and the healthy controls on shame scale of TOSCA (*t*_(1, 133)_ = 0.53, *p*<.96). As the groups differed in terms of age and gender, we also analyzed between group differences using a linear regression model controlling for age and gender. The results of this analysis showed no significant between group effect of adjusted betas (*b* = 0.95, *t* = 0.55, *p*<.58). On the guilt scale of TOSCA the controls had significantly higher scores compared to participants with SAD (*t*_(1, 133)_ = 4.33, *p*<.001). The same effects on guilt were found when testing between group differences while adding age and gender as covariates (*b* = −2.50, *t* = 2.40, *p*<.02).

**Table 2:** Means, SDs and effect sizes on measures of shame, guilt, social anxiety and depressive symptom.

					Effect size	Effect size
Measure	Group	Baseline	Follow-up	P-value	Between	Within
(Scale range)		M (SD)	M (SD)		Baseline(95% CI)	Basline-post(95% CI)
**TOSCA Shame**	SAD	45.3(9.5)	41.2(9.0)	Within group, <.02*		0.44(0.10–0.78)
(5–75)				SAD vs. HC-M<.96	SAD vs. HC-M0.03(−0.20–0.37)	
	HCMain sample	45.0(8.4)				
				SAD vs. HC-R<.02*	SAD vs. HC-R 0.62(0.12–1.10)	
	HC Replication	39.7(7.6)				
**TOSCA Guilt**	SAD	54.9(6.9)	55.8(7.3)	Within group,<.43		−0.13(−0-46–0.21)
(5–75)				SAD vs. HC-M<.001**	SAD vs HC-M−0.69(−1.03–−0.35)	
	HCMain sample	59.4(6.1)				
				SAD vs. HC-R<.83	SAD vs. HC-R−0.05(−0.53–0.44)	
	HCReplication	55.2(5.2)				
**LSAS-SR**	SAD	74.7(22.0)	44.5(24.6)	Within group, <.001**		1.29(0.91–1.66)
(0–144)						
**SIAS**	SAD	47.9(16.1)	32.3(17.1)	Within group, <.001**		0.94(0.58–1.29)
(0–80)						
**BDI**	SAD	11.8(7.8)	6.7(7.3)	Within group, <.001**		0.68(0.32–1.02)
(0–63)						

Abbreviations: TOSCA, Test of Self-Conscious Affect; LSAS-SR, Liebowitz Social Anxiety Scale-Self report; SIAS, Social Interaction Anxiety Scale; BDI, Beck Depression Inventory, Post, Post-treatment; SAD, Social anxiety disorder; HC-M, Healthy controls-main sample; HC-R, Healthy controls-replication sample. Note: all between group analyses conducted on baseline scores; follow-up scores collected at one-year follow-up;

* = significant at <.05;

** = significant at <.01.

#### SAD vs. Healthy controls replication sample

The results from the analyses comparing SAD participants with the healthy control replication sample yielded a different picture. Healthy control participants in this cross validation sample had significantly lower scores on the TOSCA shame scale than those with SAD (*t*_(1, 82)_ = 2.50, *p*<.02). As displayed in Table 2 there were minimal and non-significant differences between participants with SAD and healthy controls in the replication sample in terms of guilt as measured by the TOSCA (*t*_(1, 82)_ = 0.22, *p*<.83).

### Correlations of shame, guilt, social anxiety and depression and the effect of CBT

Table 3 entails the intercorrelation matrix of measures of shame, guilt, social anxiety, and depressive symptoms for the SAD participants. As shown in Table 3, shame was significantly correlated with social anxiety and depressive symptoms as measured by the SIAS, LSAS-SR and BDI, respectively. To investigate whether depressive symptoms and social anxiety were uniquely associated with shame partial correlations were conducted controlling for depressive symptoms and social anxiety, respectively. The results showed that SIAS and BDI remained significantly correlated with the TOSCA shame scale suggesting that social anxiety and depressive symptoms are independently related to shame.

**Table 3:** Intercorrelations on measures of shame, guilt, social anxiety and depressive symptoms for participants with SAD.

Measure	Correlations(Pearson, zero-order)					Partial correlations^a^	
						TOSCA Shame	TOSCA Guilt
	1.	2.	3.	.4	5.		
**1. SIAS**	-					.29*	.17
**2. LSAS-SR**	.68**	-				.14	.09
**3. BDI**	.32**	.28*	-			.34**	.21
**4. TOSCA Shame**	.39**	.30*	.42**	-			
**5. TOSCA Guilt**	.25	.17	.28*	.61**	-		

Abbreviations: TOSCA, Test of Self-Conscious Affect; LSAS-SR, Liebowitz Social Anxiety Scale-Self report; SIAS, Social Interaction Anxiety Scale; BDI, Beck Depression Inventory. Note:

a partial correlations represent associations between TOSCA and social anxiety scales controlling for BDI scores and vice versa;

* = significant at <.05;

** = significant at <.01. All correlations are based on data collected before treatment.

Within group t-tests showed that participants with SAD significantly reduced their shame as assessed with the TOSCA shame scale at follow-up compared to baseline (*t*_(43)_ = 2.62, *p*<.02). At follow-up participants in the SAD sample had significantly lower scores on the TOSCA shame scale compared to controls in the main sample (*t*_(1, 133)_ = 4.33, *p*<.001). Analysis of the SAD sample after CBT and the healthy control replication sample revealed that there was no longer a significant difference between the groups (*t*_(1, 68)_ = 0.64, *p*<.53). In short, in comparison with the main control sample, participants with SAD had similar shame scores at baseline but lower scores at follow-up, but in comparison with the control replications sample they had higher levels of shame at baseline but similar levels of shame after treatment with CBT. No significant change from baseline to follow-up was found on the guilt subscale (*t*_(43)_ = −0.79, *p*<.43).

As reported in the article of the original RCT [31] and shown in Table 2 participants made significant improvements from baseline to follow-up on measures of social anxiety and depressive symptoms, i.e. on the SIAS, LSAS-SR, and the BDI (*t*_(60)_ = 4.7–9.1, *p*<.001).

### Effect of treatment format on the association of shame and social anxiety

As the participants with SAD were randomized to receive CBT in an individual format or in a group format, we also investigated whether mode of delivery moderated the association between shame and social anxiety. Intriguingly, there was a close to significant correlation between shame change scores and change in social anxiety as assessed with the LSAS-SR among participants receiving group CBT (*r* = .42, *p*<.06), whereas there was a non-significant negative correlation in the individual CBT sample (*r* = −.17, *p*<.47). An additional finding was that baseline shame predicted pre-to post-treatment change in social anxiety as assessed with LSAS-SR, i.e. higher shame scores predicted better outcome, among participants receiving group treatment (*r* = .42, *p*<.02), while there was no association between baseline shame and improvement in the group receiving individual treatment (*r* = −.08, *p*<.67). Thus, shame played a role both as predictor of outcome and as a variable that followed the change pattern of social anxiety among participants with SAD in the group CBT. However, shame had no such effects in individual CBT.

## Discussion

The aim of this study was to investigate the interrelations of shame, guilt, social anxiety, and depressive symptoms by comparing persons with SAD with healthy controls, and by investigating the effect of CBT for SAD on shame and guilt. The results showed a significant association of shame and social anxiety among participants with SAD, although there were conflicting results in terms of shame between groups, where the SAD sample had elevated shame in comparison to the healthy control replication sample, but not to the main control sample. As expected, CBT led to significantly lower levels of shame. In accordance with our hypothesis, guilt was unrelated to social anxiety both in terms of lack of difference between the SAD and the healthy control replication sample, and as indicated by the finding that there was no significant correlation between guilt and social anxiety within the SAD sample. However, there was a significant difference in guilt between the SAD and the main control sample, but this was in the opposite direction as controls had higher levels of guilt than participants with SAD.

The results of this study suggest that the association between shame and social anxiety is fairly complex. First, it is quite clear that shame and social anxiety are not interchangeable concepts, which was not least demonstrated in the fact that there were minimal differences between SAD participants and healthy controls in the main sample. Nevertheless there was an association between social anxiety and shame within the SAD sample. How are these results to be understood? We believe that the differences between the two samples of healthy controls shed some light on these findings. When a new sample of controls was recruited that better matched the clinical sample a quite different picture emerged. In comparison to the replication control group the levels of shame were significantly elevated in the SAD group, and after effective treatment with CBT, the clinical sample was similar to the healthy control replication sample in terms of shame. This might reflect a true age effect on internal shame, but could also be an artifact driven by the construction of the TOSCA scale. To a large extent the potential shameful situations that are rated relate to the work place environment and it could be that persons with more work experience, as in the replication sample, have a better understanding of this context while students to some degree must “guess” what it would be like to be in the described situations. A strength in the study design was that both control samples were recruited among psychology/psychotherapist students, with the difference that many of the replication students were licensed psychologists in training to become psychotherapists. This means that the samples are likely to be recruited from the same student populations but from different time cohorts. That is, had this study been conducted 10 years earlier the participants in the replication sample would have been part of the main sample. Taken together this probably indicates that replication sample is a more valid control group. It is also worth noticing that a significant effect between the clinical sample and the replication sample in terms of shame was found despite power being reduced due to the smaller n of this control group, suggesting an at least moderately large between- group difference. Another important aspect to bear in mind when interpreting the findings of this study is that the TOSCA primarily assesses internal shame and not external. This means that the type of shame investigated pertains not the typical aspect of shame that one believes that others have negative perceptions about one-self, but concerns a more profound form of shame that is more related to self-perception. That is, it might be that shame is related to social anxiety both among persons with SAD and healthy controls but that protective factors among healthy controls reduce the effect of shame on social anxiety.

As expected, participants with SAD reduced their internal shame following CBT. This suggests that although internal shame is not explicitly targeted in CBT, the treatment affects processes relating to internal shame. It might be that skills acquired to challenge dysfunctional beliefs are used also to dispute thoughts concerning self-worth. That is, components aimed at reducing external shame are also used to reduce internal shame. Treatment modality moderated the effect of shame as a predictor and to which extent it was related to reduction of social anxiety. For participants receiving group CBT shame was associated with outcome, but no such effects were found among participants in individual therapy. A possible interpretation of these findings is that exposure to other persons with SAD has a large therapeutic impact on those with high internal shame as they become aware that other people have the same social fears. A clinical implication of these finding could be that group CBT is especially suitable for persons with SAD who have high levels of internal shame. Of course, these findings need to be replicated. A limitation concerning these findings is that a repeated measurements within-group design was used meaning that causality of CBT on the reduction of shame is uncertain. That is, it cannot be ruled out that a change in shame would have occurred also in the absence of treatment with CBT. However, as SAD as well as shame seem to be stable over time in the absence of treatment [33], [34], [35], we regard it as likely that change in shame and social anxiety was related to the treatment received. Future studies should investigate whether this effect is specific for CBT or if it is found also in other psychological and pharmacological treatments of SAD.

When comparing the degree of shame in this study to estimates found in other studies, it is just about in the same range as in the study by Fergus et al. [11] in which a clinical sample of patients with anxiety disorders were investigated using the TOSCA and slightly lower than in a study investigating persons with depression [7]. Interestingly, the healthy controls in the latter study was comprised of a sample of university students as in the present study and their shame scores were nearly identical to the scores of the main control group in this study. Once again, as shame scores were significantly lower in the replication sample of our study, this might suggest that young students are not the best matches for typical adult clinical samples.

In this study, social anxiety and depressive symptoms were shown to be independently related to shame, i.e. the association between social anxiety could not be accounted for by shared variance with depressive symptoms, and vice versa. This was the case if using the SIAS as measure of social anxiety but not if using the LSAS-SR. A possible explanation for this difference in results between the scales is that SIAS has a stronger emphasis on the emotional response, i.e. anxiety, to social stimuli whereas the LSAS-SR to an equal extent measures behavioral avoidance and the emotional response. The finding that social anxiety and depressive symptoms were independently related to shame is slightly different compared to some previous research [11] that has suggested that correlation between depressive symptoms and shame could be explained by anxiety. Our finding could suggest that in order to reduce shame in the treatment of SAD it is important to address social fears as well as depressive symptomatology.

As for guilt, the results were in line with our hypothesis that this construct would be unrelated to social anxiety. As stated above, the only statistically significant finding regarding guilt was that it was elevated in the main control sample compared to participants with SAD. These findings are similar to those obtained in previous studies [7], [11] and add to the body of knowledge indicating that guilt is largely a non-pathogenic adaptive quality that fosters salutogenic restorative behaviors. As described by Gilbert [7] the TOSCA is a good instrument to assess guilt as it clearly defines its specific characteristics vis-à-vis shame, e.g. focus on harm done and concern for others' suffering. This reduces the risk of losing precision as shame and guilt in everyday language are often confused, which could blur findings if participants are simply asked to report on feelings of guilt without further specification.

A general note when interpreting the findings of the present paper was that the study was carried out in a western-world context. As it has been shown that the significance of shame on anxiety in non-clinical samples could be moderated by ethnicity [36], caution is warranted in terms of generalizability of the results to other cultural contexts.

This study has some limitations. First, patients with SAD and healthy controls in the main sample were not matched on age and gender, which could have biased the results. However, the inclusion of a replication sample that better resembled the clinical sample on demographic characteristics enabled a more comprehensive analysis. A second limitation was the relatively small sample size reducing the power to detect small differences between groups. There was however adequate power to detect a difference of moderate size and it can be argued that smaller differences are of less clinical importance.

In spite of these limitations we regard the findings of the present study as important as they demonstrate that internal shame, social anxiety and SAD are associated and that shame is likely to be elevated in persons with SAD compared to healthy controls. In addition, to our knowledge this study is the first to demonstrate that CBT effectively reduces internal shame in the treatment of SAD.
